# Endoplasmic Reticulum Stress Activates the Hepatic Activator Protein 1 Complex via Mitogen Activated Protein Kinase-Dependent Signaling Pathways

**DOI:** 10.1371/journal.pone.0103828

**Published:** 2014-07-31

**Authors:** Shantel Olivares, Richard M. Green, Anne S. Henkel

**Affiliations:** Division of Gastroenterology and Hepatology, Northwestern University Feinberg School of Medicine, Chicago, Illinois, United States of America; UAE University, Faculty of Medicine & Health Sciences, United Arab Emirates

## Abstract

**Background and Aims:**

Endoplasmic reticulum (ER) stress is induced in many forms of chronic liver disease and may promote the development of hepatocellular carcinoma. The activator protein 1 (AP-1) complex is a transcription factor that promotes hepatic carcinogenesis in response to cellular stress. The aim of this study was to determine the role of ER stress in the regulation of the hepatic AP-1 complex.

**Methods:**

Human hepatocellular carcinoma (HepG2) cells and C57BL/6J mice were subjected to pharmacologic ER stress and the expression of AP-1-associated genes and proteins was assessed. To determine the role of MAPK signaling in ER stress-induced AP-1 activation, ER stress was induced in JNK- and ERK-inhibited HepG2 cells.

**Results:**

Induction of ER stress promoted the activation of both Jun- and Fos-related genes and proteins of the AP-1 complex in HepG2 cells and murine liver. Inhibition of ERK phosphorylation in HepG2 cells completely prevented ER stress-induced activation of the fos-related components of AP-1 whereas activation of Jun-related components was only partially attenuated. Conversely, inhibition of JNK phosphorylation in HepG2 cells reduced ER stress-induced activation of Jun-related components but did not prevent activation of fos-related components.

**Conclusions:**

ER stress activates the hepatic AP-1 complex via MAPK-dependent signaling pathways. ER stress-induced activation of Fos-related components is dependent primarily on ERK activation whereas ER stress-induced activation of Jun-related components is dependent primarily on JNK activation, although there is interplay between these regulatory pathways. These data implicate a novel signaling pathway by which sustained ER stress, as observed in many chronic liver diseases, may promote hepatic carcinogenesis.

## Introduction

The incidence of hepatocellular carcinoma (HCC) has more than doubled over the past 20 years attributable in large part to the high rate of new hepatitis C virus infection 30 to 40 years ago [1], [2]. Cirrhosis is the primary risk factor for HCC, however, the underlying cause of cirrhosis impacts the risk of progressing to HCC. It is becoming increasingly evident that patients with cirrhosis secondary to nonalcoholic fatty liver disease (NAFLD) are at particularly high risk for developing HCC. In fact, recent observations suggest that NAFLD is a risk factor for HCC even in the absence of cirrhosis [3], [4]. Although significant progress is being made toward curing chronic hepatitis C, the prevalence of NAFLD is rapidly escalating in the United States. As such, the burden of HCC is unlikely to decline in the foreseeable future [5], [6].

Endoplasmic reticulum (ER) stress and the ensuing unfolded protein response are strongly implicated in the pathogenesis of many forms of chronic liver disease, including hepatitis C infection, alcoholic liver disease, and NAFLD [7]–[14]. The ER functions to maintain protein homeostasis by regulating protein synthesis, folding and processing. Under conditions of ER stress, such as glucose deprivation, aberrant calcium signaling, viral infection, lipotoxicity, and disruption of redox regulation, normal ER function becomes compromised leading to the accumulation of unfolded or misfolded proteins [15]. The accumulation of unfolded proteins triggers an evolutionarily conserved intracellular signal transduction pathway known as the unfolded protein response (UPR) [16]. The UPR initially aims to restore homeostasis and allow the cell to adapt to the stressor [17]. If homeostasis is not adequately restored, however, pathways leading to apoptosis are initiated [18], [19]. Mitogen activated protein kinases (MAPKs) are activated in response to ER stress and may mediate, in part, the critical switch from restoration of homeostasis to initiation of apoptosis. In particular, cJun N-terminal kinase (JNK), a well-established downstream target of the IRE1α branch of the UPR, is thought to promote ER stress-induced apoptosis [20], [21]. The role of extracellular signal-regulated protein kinase (ERK) activation in mediating the ER stress response is less well-characterized but may function to enhance cell survival [22], [23]. The mechanism by which ER stress induces ERK is incompletely understood but is thought to be at least partially mediated by PI3K and adaptor protein Nck [22], [23].

Recent data suggest that ER stress and the UPR may be important in the development of hepatocellular carcinoma [24]–[31]. Grp78/BiP, a molecular chaperone and master regulator of the UPR, has been posited to promote malignant transformation in numerous tissues including the liver [32]–[34]. ER stress has also been implicated in the pathogenesis of liver cancer resulting from chronic alcohol use [35]. Sorafenib, the only approved chemotherapeutic agent to treat HCC, has been shown to modulate the UPR [26], [27]. Yet, it remains unknown whether induction of ER stress contributes to the development of HCC in chronic liver diseases.

Activation of the activator protein 1 (AP-1) complex has been shown to be a critical event in the development of HCC [36]. The AP-1 complex is a dimer composed of protooncogenes from the Jun family (*cJun*, *JunD*, and *JunB*) and the Fos family (*cFos*, *FosB*, *Fra-1*, and *Fra-2*) [37], [38]. In response to various types of cellular stress, the AP-1 complex is activated to function as a transcription factor targeting genes involved in cellular differentiation, proliferation, and apoptosis [37], [38]. MAPKs are key regulators of numerous protooncogenes, including elements of the AP-1 complex [39], [40].

In the present study we aim to determine the impact of ER stress on the regulation of the hepatic AP-1 complex. Specifically we will test our hypothesis that ER stress activates the hepatic AP-1 complex via activation of MAPK-dependent signaling pathways.

## Materials and Methods

### Cell culture

Human hepatocellular carcinoma (HepG2) cells (ATCC, Mannasas, VA) were cultured in DMEM with 10% fetal bovine serum and maintained at 37°C in 5% CO_2_. Cells were grown to 80% confluence in 6-well plates and treated with 12 µM tunicamycin, 100 nM thapsigargin (Sigma-Aldrich, St. Louis, MO), 5 mM DL-homocysteine (Sigma-Aldrich) or vehicle (DMSO/saline) in serum-free DMEM for 6 hours. RNA isolation was performed using TRIzol Reagent (Invitrogen, Carlsbad, CA) per protocol. To determine whether the effects of tunicamycin are dependent on ERK1/2 signaling, HepG2 cells were treated with the MEK inhibitor, PD184352 (Santa Cruz Biotechnology) at a concentration of 1 µM or vehicle (DMSO/saline). To determine whether the effects of tunicamycin are dependent on cJun-N-terminal kinase (JNK) signaling, HepG2 cells were treated with the JNK inhibitor, SP600125 (Sigma-Aldrich) at a concentration of 25 µM. After one hour of exposure to PD184352 or SP600125, cells were treated with tunicamycin (10 µg/mL) (Sigma-Aldrich) in serum-free DMEM and incubated for an additional 6 hours. Cytotoxicity was assessed by measuring LDH release in cell culture media using a Cytotox 96 Nonradioactive Cytotoxicity Assay (Promega, Madison, WI). Cellular proliferation was assessed using a BrdU Cell Proliferation ELISA kit (Abcam, Cambridge, MA). In a separate experiment, cells were treated with siRNA targeting *JNK1* or control siRNA (Santa Cruz Biotechnology). HepG2 cells grown to 80% confluence were treated with *JNK1* siRNA or control siRNA per protocol. Briefly, for each transfection, 6 µL of control or *JNK1* siRNA were diluted in 100 µL of siRNA Transfection Medium (Solution A) and 6 µL of siRNA Transfection Reagent was diluted in 100 µL of siRNA Transfection Medium (Solution B). Fifty microliters of Solutions A was added to 100 µL of Solution B and allowed to incubate at room temperature for 30 minutes. After washing the cells once with Transfection Medium, 0.8 mL of the siRNA in Transfection Reagent was added to each well. Cells were incubated for 6 hours at 37°C. The transfection medium was then removed, the cells were washed and incubated in normal growth medium (DMEM with 10% FBS) for 18 hours. The cells were then treated with tunicamycin (10 µg/mL) (Sigma-Aldrich) in serum-free DMEM for 6 hours. The doses of the inhibitors/siRNA were experimentally determined to achieve near complete inhibition of ERK- and JNK-phosphorylation with PD184352 and SP600125, respectively, and an approximately 50% inhibition of JNK1 using JNK1 siRNA as confirmed by western blot (Figure S1).

### Animals and Treatments

Male C57BL/6J mice (8 weeks of age) were purchased from Jackson Laboratories (Bar Harbor, ME). Mice underwent 14/10-hour light/dark cycling and were given free access to standard laboratory chow and water. Mice were treated with a single intraperitoneal injection of tunicamycin (2 mg/kg) or vehicle (20% DMSO/PBS) and sacrificed 6 or 72 hours post-injection. To examine the effects of prolonged, sublethal exposure to ER stress, an additional cohort of mice was treated with a daily injection of low-dose tunicamycin (0.1 mg/kg I.P.) for 5 days to achieve a cumulative dose of 0.5 mg/kg. At the end of the treatment protocols, mice were sacrificed by CO2 inhalation followed by cardiac puncture. The collected blood was immediately centrifuged to collect the plasma. The livers were rapidly excised, flushed with ice-cold saline and sectioned. An aliquot of liver was fixed in formalin and the remaining liver was snap-frozen in liquid nitrogen. H&E staining, TUNEL staining, and immunohistochemical staining for Ki67 were performed on liver tissue by the Northwestern University Pathology Core. Plasma ALT was measured using a colorimetric assay per manufacturer's protocol (Teco Diagnostics, Anaheim, CA). All animal protocols were approved by the Northwestern University Animal Care and Use Committee (ACUC).

### Analysis of Gene Expression and Protein expression

Total RNA from frozen liver samples or HepG2 cells was isolated using TRIzol reagent and real-time quantitative PCR was performed as previously described [11], [41]. Primer sequences for qPCR are shown in Table S1. Total protein was isolated from frozen liver samples and HepG2 cells and Western blotting was performed as previously described [11], [41]. Protein detection was performed using polyclonal rabbit antibodies to total and phosphorylated cFos, cJun, ERK, and JNK (Cell Signaling Technology, Danvers, MA). Bound antibody was detected using goat anti-rabbit polyclonal HRP antibody (Cell Signaling Technology) and developed using ECL Western Blotting Substrate (Cell Signaling Technology). Representative Western blots of pooled samples are shown.

### Statistical Analysis

Data are presented as mean ± standard deviation (SD). Comparisons between groups were performed using Student's *t*-test analysis.

## Results

### Endoplasmic reticulum stress activates AP-1-associated genes in human hepatocellular carcinoma cells

**Figure 1:**
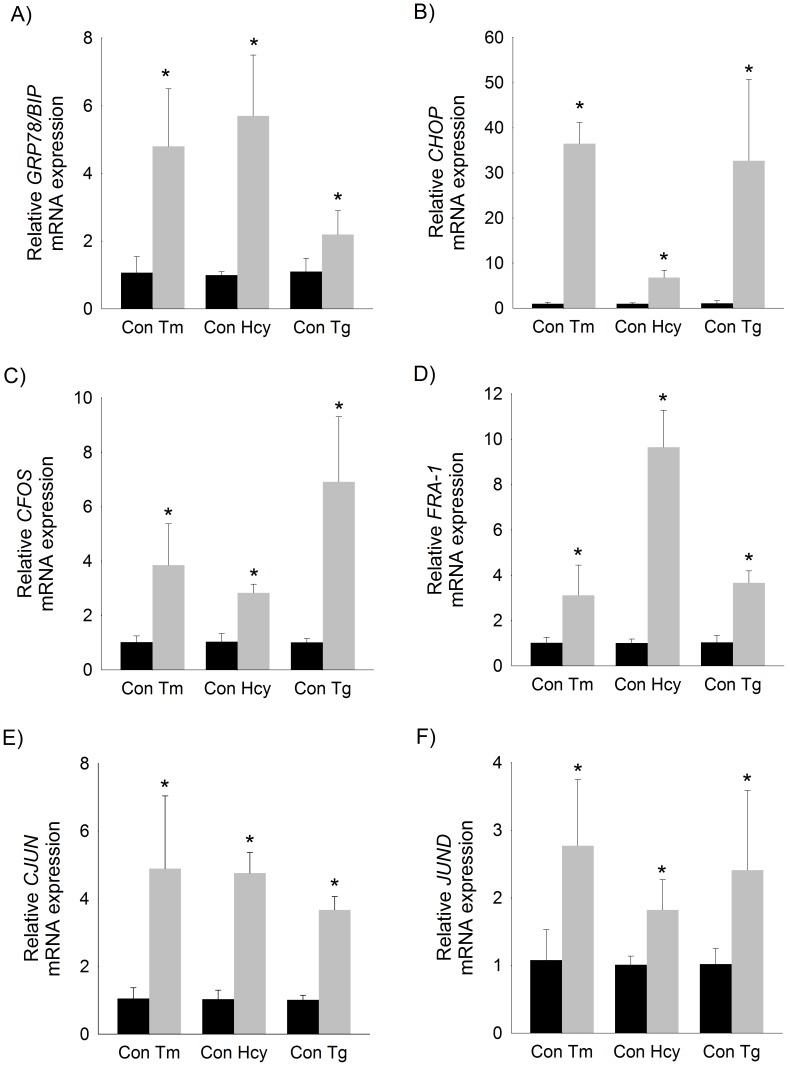
Induction of ER stress transcriptionally activates genes of the AP-1 complex in HepG2 cells. Relative mRNA expression of A) *GRP78/BIP*, B) *CHOP*, C) *CFOS*, D) *FRA-1*, E) *CJUN*, and F) *JUND* in HepG2 cells treated with tunicamycin (Tm), homocysteine (Hcy) or thapsigargin (Tg) for 6 hours. Mean (n = 6) ± SD. * p<0.05 vs vehicle-treated control cells.

To determine the effect of ER stress on activation of the AP-1 complex, human hepatocellular carcinoma cells (HepG2) were treated with three well-established ER stress inducing agents, tunicamycin, thapsigargin, and homocysteine. As expected, all treatments resulted in robust activation of the unfolded protein response as evidenced by elevations in *GRP78/BIP* and *CHOP* (Figure 1 A,B). We then examined the effect of ER stress on the expression of the fos-related genes (*CFOS* and fos-related antigen 1 (*FRA1*)), and jun-related genes (*CJUN* and *JUND*) of the AP-1 complex. As shown in Figure 1, induction of ER stress by any of the three pharmacologic methods stimulated transcription of both Jun- and Fos-related genes of the AP-1 complex.

### Endoplasmic reticulum stress activates AP-1 in murine liver

**Figure 2:**
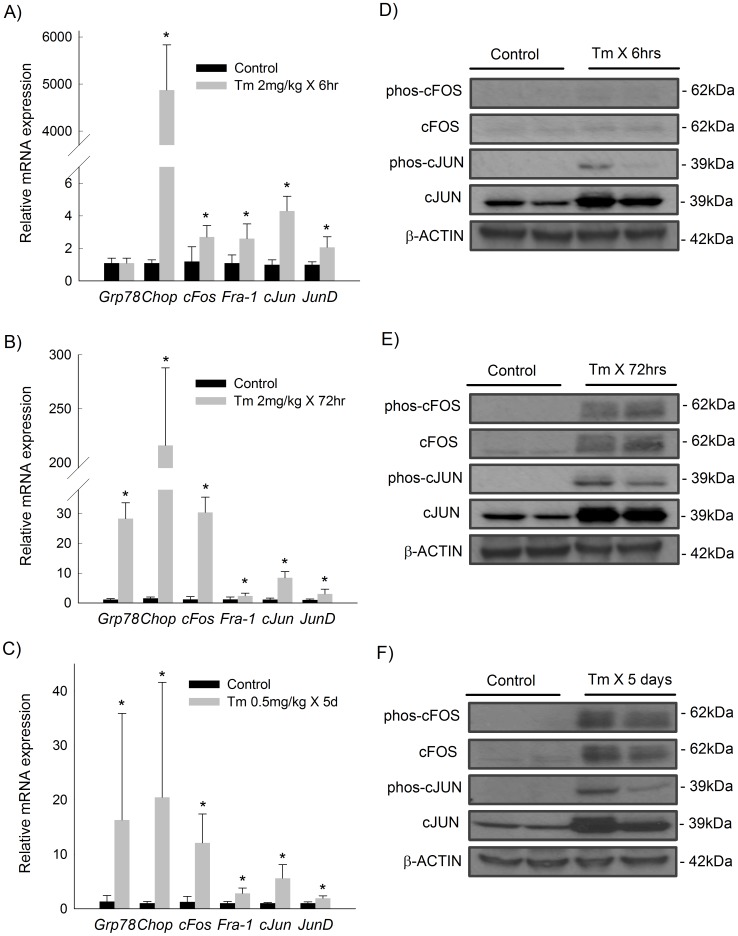
Induction of ER stress in mice activates the hepatic AP-1 complex. Relative hepatic mRNA expression of G*rp78/Bip*, *Chop*, *cFos*, *Fra-1*, *cJun*, and *JunD* in mice treated with tunicamycin (Tm) at doses of A) 2 mg/kg for 6 hours, B) 2 mg/kg for 72 hours, or C) 0.5 mg/kg over 5 days. Hepatic protein expression of total and phosphorylated CFOS and CJUN in mice treated with Tm at doses of D) 2 mg/kg for 6 hours, E) 2 mg/kg for 72 hours, or F) 0.5 mg/kg over 5 days. Quantitative PCR shown as mean (n = 6) ± SD. * p<0.05 vs vehicle-treated. Representative Western blot of pooled samples, n = 2–3.

We next sought to determine whether ER stress activates the AP-1 complex *in vivo*. Mice were treated with a standard dose of tunicamycin (2 mg/kg I.P.) and sacrificed 6 or 72 hours later. Tunicamycin treatment resulted in significant induction of the unfolded protein response in murine liver at both time points (Figure 2 A,B). There was significant induction of hepatic *cFos*, *Fra-1*, *cJun*, and *JunD* mRNA 6 hours after exposure to tunicamycin (Figure 2A). The AP-1-associated genes were induced to an even greater degree 72 hours after tunicamycin administration (Figure 2B).

A standard experimental dose of tunicamycin (2 mg/kg) induces overwhelming ER stress from which mice are unable to restore homeostasis and do not typically survive beyond 4 days. Chronic liver diseases, however, induce low-grade ER stress over prolonged periods. Therefore, to examine the effects of prolonged, sublethal exposure to ER stress, mice were treated with daily low-dose injections of tunicamycin over 5 days to achieve a cumulative dose of 0.5 mg/kg. Robust UPR activation was achieved with this dosing strategy (Figure 2C). Mice subjected to prolonged, low-dose tunicamycin showed induction of both fos- and jun-related genes of the AP-1 complex (Figure 2C).

The AP-1 complex is also subject to regulation at the translational and post-translational levels. As shown in Figure 2, hepatic CJUN was consistently increased at the translational and post-translational level in response to 6 hours, 72 hours, or 5 days of ER stress. Total and phosphorylated CFOS were only faintly detectable at 6 hours but did demonstrate modestly enhanced expression with exposure to tunicamycin (Figure 2D). At the 72-hour and 5-day time points we observed greatly increased levels of both total and phosphorylated CFOS in response to ER stress (Figure 2 E,F).

**Figure 3:**
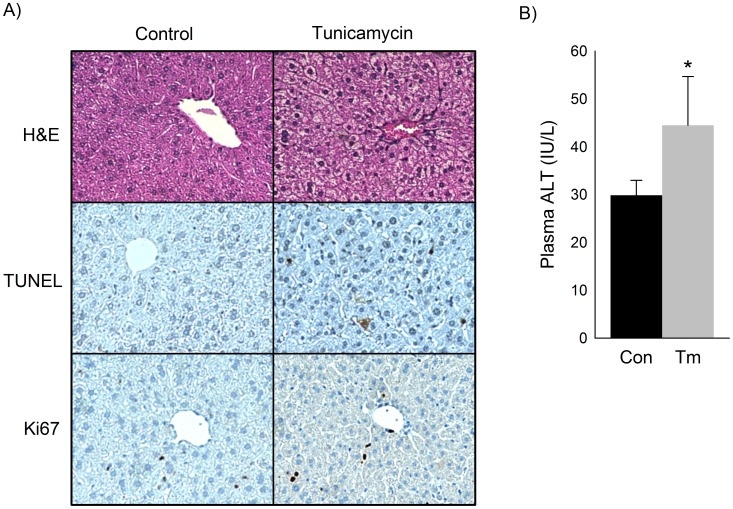
Prolonged low-grade ER stress promotes hepatocyte injury and apoptosis. Markers of liver injury, apoptosis, and cellular proliferation in mice treated with a cumulative dose of 0.5/kg tunicamycin over 5 days. A) Representative H&E, TUNEL, and Ki67 staining of liver sections. B) Plasma ALT level (IU/L). Mean (n = 6) ± SD, *p<0.05.

ER stress and AP-1 activation have been shown to promote both apoptosis and cellular proliferation. We found that mice exposed to low-dose tunicamycin for 5 days developed hepatocyte ballooning and increased apoptosis in the liver but no significant increase in cellular proliferation (Figure 3A). Plasma ALT, a common marker of hepatic injury, was modestly increased in mice treated with tunicamycin (Figure 3B).

### Induction of Fos-related components of AP-1 by ER stress is dependent on ERK activation

**Figure 4:**
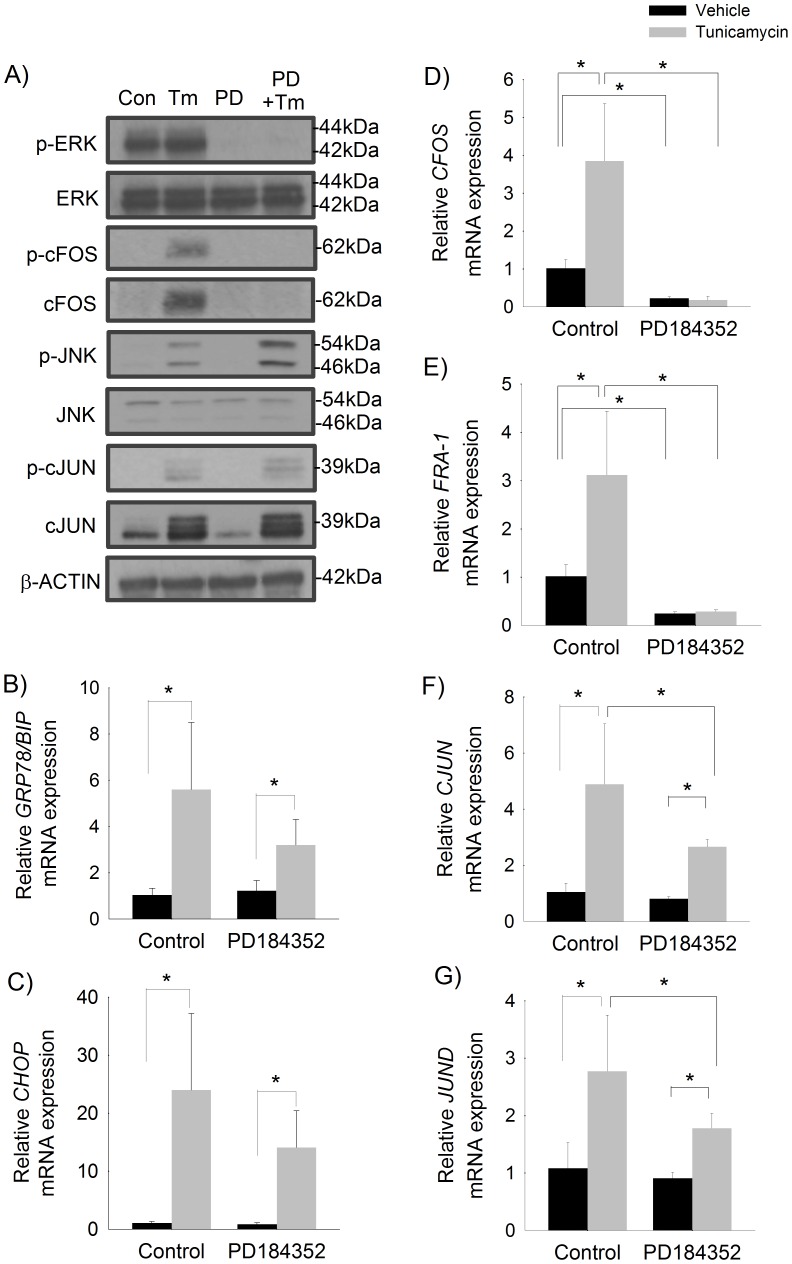
ER stress-induced activation of *CFOS* and *FRA-1* is mediated by ERK1/2 signaling. HepG2 cells pretreated with MEK1 inhibitor (PD) or vehicle followed by treatment with tunicamycin (Tm) or vehicle (control) for 6 hours. A) Representative Western blot of total and phosphorylated ERK, JNK, CFOS, and CJUN. Relative mRNA expression of B) *GRP78/BIP*, C) *CHOP*, D) *CFOS*, E) *FRA-1*, F) *CJUN*, and G) *JUND*. Mean (n = 6) ± SD. * p<0.05.

We next sought to determine the mechanism by which ER stress activates the AP-1 complex in human hepatocellular carcinoma cells. ER stress has been shown to activate MAPK signaling pathways [20], [21], [23]. Furthermore, ERK activation is known to promote the transcription of fos-related genes [42], [43]. We therefore explored whether ER stress activates AP-1-associated genes via an ERK-dependent signaling pathway. HepG2 cells were pretreated with the potent MEK inhibitor, PD184352, which prevents ERK1/2 phosphorylation (activation), followed by treatment with tunicamycin to induce ER stress. As shown in Figure 4, treatment with tunicamycin induced ERK phosphorylation, which was completely abolished by pre-treatment with PD184352. Inhibition of ERK phosphorylation did not prevent tunicamycin-induced upregulation of ER stress markers, *GRP78/BIP* and *CHOP*, however, there was a trend toward a reduction in the degree of upregulation of these markers (Figure 4 B,C). PD184352 completely abolished the activation of *CFOS* and *FRA-1* by tunicamycin at the transcriptional level (Figure 4 D,E). Tunicamycin-induced transcription of *CJUN* and *JUND* was attenuated by ERK inhibition (Figure 4 F,G).

We found that ERK signaling also regulates AP-1 at the translational and post-translational levels (Figure 4A). Tunicamycin increased total and phosphorylated CFOS protein levels, which was completely abolished by ERK-inhibition, thus correlating with CFOS gene expression. Conversely, inhibition of ERK phosphorylation accentuated tunicamycin-induced CJUN phosphorylation. We also found that inhibition of ERK-phosphorylation increased levels of phosphorylated JNK, a key activator of CJUN.

**Figure 5:**
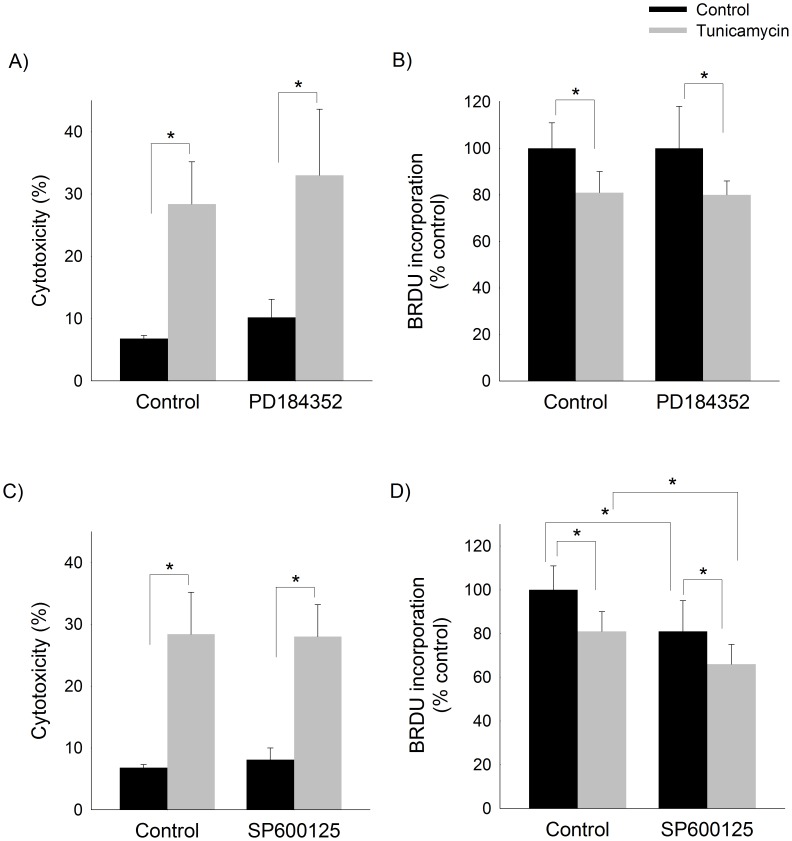
Effects of ERK- and JNK-inhibition on tunicamycin-induced cytotoxicity and cellular proliferation. Cytoxicity as measured by LDH release (A,C) and cellular proliferation as measured by BrdU incorporation (B,D) in HepG2 cells pretreated with MEK1 inhibitor (PD184352) or JNK inhibitor (SP600125) followed by treatment with tunicamycin or vehicle (control) for 6 hours. Mean (n = 6) ± SD. *p<0.05.

As expected, treatment of HepG2 cells with tunicamycin increased cytotoxicity as measured by LDH release (Figure 5A). We found that ERK-inhibition did not attenuate tunicamycin-induced cell death. Induction of ER stress inhibited cellular proliferation which was also unaffected by ERK-inhibition (Figure 5B).

### Induction of Jun-related components of AP-1 by ER stress is dependent on JNK activation

**Figure 6:**
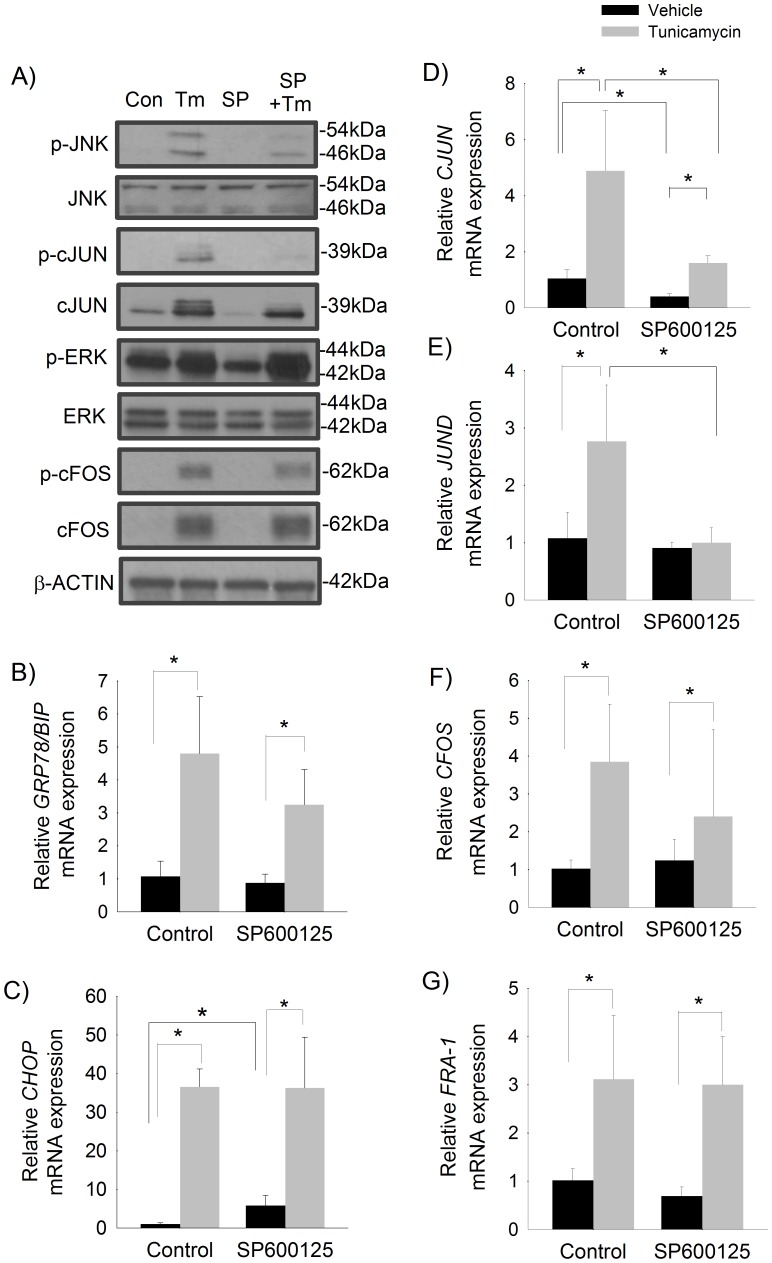
ER stress-induced activation of *CJUN* and *JUND* is mediated by JNK signaling. HepG2 cells pretreated with JNK inhibitor (SP600125) or vehicle followed by treatment with tunicamycin or vehicle (control) for 6 hours. A) Representative Western blot of total and phosphorylated JNK, ERK, CJUN, and CFOS. Relative mRNA expression of B) *GRP78/BIP*, C) *CHOP*, D) *CJUN*, E) *JUND*, F) *CFOS*, and G) *FRA-1*. Mean (n = 6) ± SD. * p<0.05.

JNK, a MAP kinase that mediates multiple stress signaling pathways in the liver, is a well-established downstream target of the ER stress response [20], [21]. Furthermore, JNK is a critical mediator of hepatic injury in fatty liver diseases and has been implicated in the development of HCC [44]–[46]. Given our observation that ER stress-induced activation of Jun-related genes is not completely abolished by inhibition of ERK phosphorylation, we next explored whether ER stress activates Jun-related genes via JNK activation. HepG2 cells were treated with the JNK inhibitor, SP600125, followed by treatment with tunicamycin. As expected, treatment of HepG2 cells with tunicamycin markedly induced JNK phosphorylation, which was attenuated by pretreatment with SP600125 (Figure 6A). Inhibition of JNK phosphorylation did not significantly impact tunicamycin-induced *GRP78/BIP* or *CHOP* activation however there was a trend toward reduced *GRP78/BIP* transcription in JNK-inhibited cells (Figure 6 B,C). Inhibition of JNK phosphorylation reduced tunicamycin-induced *CJUN* transcription and completely abolished upregulation of *JUND* transcription (Figure 6 D,E). *CFOS* and *FRA-1* expression was induced by tunicamycin in JNK–inhibited cells (Figure 6 C,D); however, the degree of *CFOS* induction was attenuated by JNK-inhibition, indicating that ER stress-induced *CFOS* elevation is at least partially mediated by JNK signaling.

As shown in Figure 6A, inhibition of JNK phosphorylation attenuated the induction of total and phosphorylated CJUN protein by tunicamycin. Conversely, ER stress-induced CFOS activation was not attenuated by JNK-inhibition. ER stress-induced ERK phosphorylation was enhanced in JNK-inhibited cells.

Similar to inhibition of ERK phosphorylation, inhibition of JNK phosphorylation did not attenuate tunicamycin-induced cytotoxicity (Figure 5C). Inhibition of JNK activation did, however, enhance the effects of tunicamycin on inhibition of cellular proliferation (Figure 5D).

**Figure 7:**
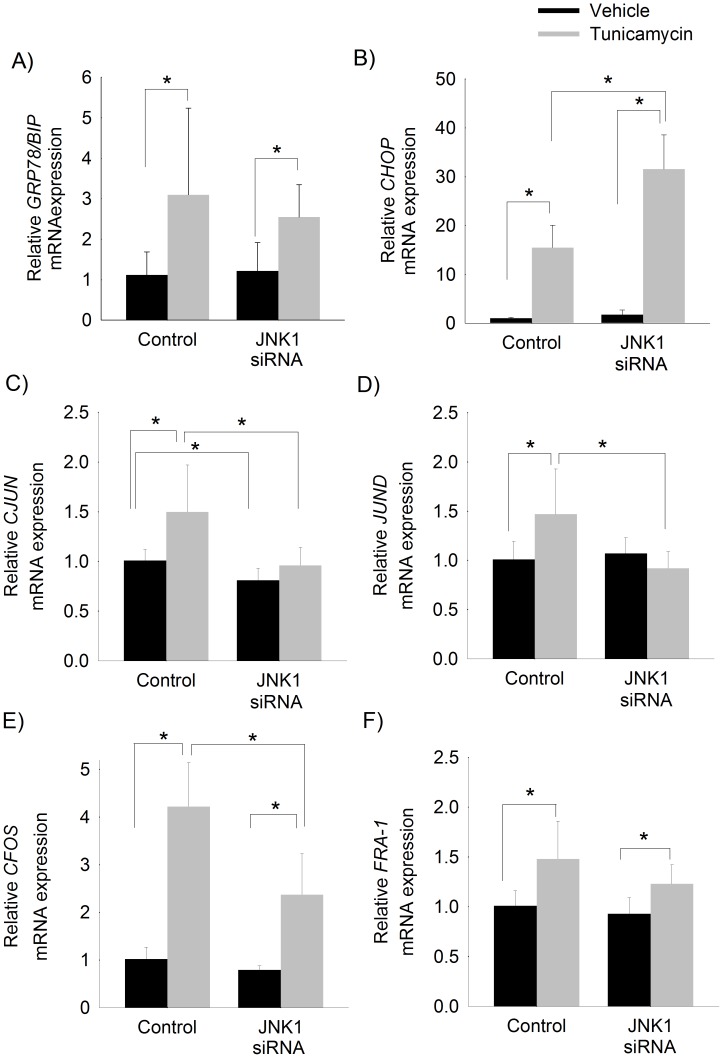
ER stress-induced transcriptional activation of *CJUN* and *JUND* is mediated by JNK1 signaling. Relative mRNA expression of A) *GRP78/BIP*, B) *CHOP*, C) *CJUN*, D) *JUND*, E) *CFOS*, and F) *FRA-1* in HepG2 cells pretreated with *JNK1* siRNA or control siRNA followed by treatment with tunicamycin or vehicle (control) for 6 hours. Mean (n = 6) ± SD. * p<0.05.

Given that sustained activation of the JNK1 isoform, in particular, has been associated with steatohepatitis and the development of HCC [44]–[46] we confirmed the findings observed in JNK-inhibited cells using JNK1-silenced cells. Silencing of JNK1 did not effect tunicamycin-induced *GRP78/BiP* transcription but accentuated *CHOP* expression (Figure 7 A,B). Silencing JNK1 prevented tunicamycin-induced transcription of *CJUN* and *JUND* but not *CFOS* or *FRA-1* (Figure 7 C–F).

## Discussion

Endoplasmic reticulum stress and the ensuing UPR are now recognized as mediators of numerous pathologic processes in the liver. The UPR is activated in several chronic liver diseases that ultimately promote the development of HCC including NAFLD, alcohol-induced liver disease and hepatitis C [7]–[13]. However, it is unknown whether activation of the UPR in these chronic liver diseases contributes to the increased risk of HCC.

The AP-1 complex is a critical regulator of hepatic carcinogenesis [36]. Here we demonstrate that induction of ER stress in human hepatocellular carcinoma cells and murine liver activates the AP-1 complex at the transcriptional, translational, and post-translational levels. We show that ER stress regulates fos-related components of the AP-1 complex primarily via activation of ERK signaling whereas the activation of jun-related components is primarily mediated by JNK. There is, however, an element of cross-talk between these pathways as evidenced by attenuated induction of *fos-*related genes in JNK-inhibited cells and attenuated induction of *jun-*related genes in ERK-inhibited cells in response to ER stress. Given the established role of AP-1 in promoting hepatic carcinogenesis, we speculate that ER stress induced AP-1 activation may be a mechanism by which chronic liver diseases promote malignant transformation.

The effect of JNK1-silencing on the expression of AP-1-associated genes paralleled that of HepG2 cells treated with SP600125 indicating that specifically the JNK1 isoform is critical for AP-1 activation. Of note, the degree of tunicamycin-induced UPR activation and resultant AP-1 gene transcription was modestly attenuated in HepG2 cells pre-treated with control (scrambled) siRNA relative to non-transfected control cells (e.g. Figures 1, 4, and 6). Although we cannot exclude the possibility that the blunted UPR was an effect of scrambled siRNA, these differences could also be due to differing cell culture conditions required for transfection (e.g. duration of serum starvation prior to tunicamycin exposure).

Mice exhibit robust activation of the unfolded protein response within 6 hours of exposure to a standard experimental dose of tunicamycin (2 mg/kg). However, beyond 72 hours this dose of tunicamycin is typically fatal. This raised the possibility that ER stress-induced AP-1 activation may occur only in the setting of overwhelming cellular stress. As such, the physiologic relevance of our observations with respect to chronic liver disease was initially uncertain. Therefore, we also subjected mice to sustained, sublethal ER stress and demonstrate robust activation of hepatic AP-1-associated genes and proteins.

AP-1 has numerous critical functions in the liver with respect to cellular differentiation, proliferation, and apoptosis. The function of AP-1 is determined in part by the relative activation of the various components of this heterodimeric complex. The UPR is a highly dynamic signal transduction pathway in which the various branches are preferentially activated depending on the degree and duration of ER stress. We show that the degree of induction of AP-1-associated genes in murine liver varies depending on the duration and severity of ER stress. As such, the degree and duration of ER stress may impact the transcriptional activity of the AP-1 complex by shifting the relative induction of ERK and JNK and the resultant induction of jun- and fos-related genes. Although we hypothesize that ER stress and the resultant AP-1 activation may promote hepatic carcinogenesis, it is also conceivable that under conditions of chronic low-grade ER stress, activation of the UPR may confer protection against carcinogenesis (e.g. via promoting apoptosis.) The duration of this experiment was insufficient to observe tumor formation in mice, however, future studies are warranted to determine whether inhibition of ER stress may protect against the development of HCC in mice.

At the chosen dose and time points we demonstrate that tunicamycin promotes hepatocyte injury, apoptosis, and cell death and inhibits cellular proliferation. Importantly we found that inhibition of MAPK signaling pathways did not prevent tunicamycin-induced activation of ER stress markers and did not attenuate ER stress-induced cytotoxicity. These findings indicate that the observed reduction in AP-1 activation in ERK- and JNK-inhibited cells is not a consequence of reduced cellular injury.

We propose that coordinated activation of ERK1/2 and JNK1 signaling pathways by ER stress promotes activation of the hepatic AP-1 complex. These data identify a novel pathway by which ER stress, a common feature of many chronic liver diseases, may promote hepatic carcinogenesis. Currently there are limited pharmacotherapeutic options to treat HCC. This work raises the possibility that targeting the unfolded protein response may have therapeutic benefit in the treatment or prevention of HCC in patients with chronic liver disease.

## Supporting Information

Figure S1 **Successful silencing of JNK1 in HepG2 cells treated with siRNA targeting JNK1.** Representative Western blot of pooled samples (n = 6).
(TIF)

Table S1 **Primer sequences for quantitative PCR.**
(DOC)
