# Supplementary material for: Endoplasmic Reticulum Stress Activates the Hepatic Activator Protein 1 Complex via Mitogen Activated Protein Kinase-Dependent Signaling Pathways
Source: PLoS One. 2014 Jul 31;9(7):e103828. doi: 10.1371/journal.pone.0103828 (PMC4117566; doi:10.1371/journal.pone.0103828)
Supplement: Table S1 — Primer sequences for quantitative PCR. (DOC) [file pone.0103828.s002.doc]

| **Table S1: Primer sequences for quantitative PCR** | | |
| --- | --- | --- |
|  | **Forward 5’-3’** | **Reverse 5’-3’** |
| **Mouse** | | |
| Gapdh | AGGTCGGTGTGAACGGATTTG | TGTAGACCATGTAGTTGAGGTCA |
| Grp78/Bip | CGA GGA GGA GGA CAA GAA GG | CAC CTT GAA CGG GCA AGA ACT |
| Chop | CTG GAA GCC TGG TAT GAG GAT | CAG GGT CAA GAG TAG TGA AGG T |
| cFos | AGG GGC AAA GTA GAG CAG CTA | CAA TCT CAG TCT GCA ACG CA |
| Fra-1 | CAC TGG ATG GTG CAG CCT | CTA GGG CTC GTA TGA CTC CTG |
| cJun | TTCCTCCAGTCCGAGAGCG | TGAGAAGGTCCGAGTTCTTGG |
| JunD | GGCGGGATTGAAACCAGGG | AGCCCGTTGGACTGGATGA |
| **Human** | | |
| GAPDH | GTCATCATCTCTGCCCCCTCTGCTG | CGACGCCTGCTTCACCACCTTCTTG |
| GRP78/BIP | GGC CAA ATT TGA AGA GCT GA | GCT CCT TGC CAT TGA AGA AC |
| CHOP | GCACCTCCCAGAGCCCTCACTCTCC | GTCTACTCCAAGCCTTCCCCCTGCG |
| CFOS | CCGGGGATAGCCTCTCTTACT | CCAGGTCCGTGCAGAAGTC |
| FRA-1 | CAGGCGGAGACTGACAAACTG | TCCTTCCGGGATTTTGCAGAT |
| CJUN | TCCAAGTGCCGAAAAAGGAAG | CGAGTTCTGAGCTTTCAAGGT |
| JUND | TCATCATCCAGTCCAACGGG | TTCTGCTTGTGTAAATCCTCCAG |
